# Photo-supported conversations about well-being (BeWell^TM^) for patients with exhaustion disorders – a controlled clinical intervention study

**DOI:** 10.1080/02813432.2024.2421588

**Published:** 2024-11-06

**Authors:** A. Birgitta Gunnarsson, Petra Wagman, Ulrica Hörberg, Kristina Holmgren, Sara Holmberg

**Affiliations:** a Institute of Neuroscience and Physiology, Department of Health and Rehabilitation, The Sahlgrenska Academy, University of Gothenburg, Gothenburg, Sweden; b Department of Research and Development, Region Kronoberg, Växjö, Sweden; c Department of Rehabilitation, School of Health and Welfare, Jönköping University, Jönköping, Sweden; d Department of Health and Caring Sciences, Faculty of Health and Life Sciences, Linnaeus University, Växjö, Sweden; e Department of Medicine and Optometry, Faculty of Health and Life Sciences, Linnaeus University, Kalmar, Sweden; f Division of Occupational and Environmental medicine, Department of Laboratory Medicine, Lund University, Lund, Sweden

**Keywords:** Activities in everyday life, health promotion, intervention, mental health, photographs, questionnaires, Clinical Trials.gov: NCT04832295, retrospectively registered 2 April 2021 https://clinicaltrials.gov/ct2/show/NCT04832295

## Abstract

**Introduction:**

Health-promotion approaches to address stress-related exhaustion disorders, reduce personal suffering, improve coping and participation in everyday life are needed in primary care. The aim of this study was to investigate self-reported health and well-being before and after an intervention focusing on well-being with photo-supported conversations (BeWell^TM^).

**Material and methods:**

Eighty-one patients (69 women), 20–67 years old, with exhaustion disorders were recruited at Swedish primary health care centres (PHCC) to a controlled clinical study. The intervention group (*n* = 40) were offered BeWell^™^ by therapists in addition to care as usual. Controls (*n* = 41) received only care as usual. The primary outcome, self-rated symptoms of exhaustion (Karolinska exhaustion disorder scale, KEDS), and secondary outcomes, anxiety and depression, sense of coherence, quality of life, occupational balance, and work ability, were assessed by validated questionnaires. Non-parametric statistical analyses were used to compare data collected directly after the treatment period with baseline measures.

**Results:**

Demographics and self-rated baseline measures of health and well-being were comparable between the groups, apart from sick leave being more common in the intervention group. Participants in the intervention group reduced their level of exhaustion more than the control group (median difference on KEDS −9.0 vs −4.0, *p* = .035). However, the size of the KEDS reduction was related to baseline KEDS and, not independently associated with group assignment. Both groups improved regarding secondary outcome measures.

**Conclusion:**

Stress-related symptoms decreased considerably over the treatment period for both groups. The potential benefit of the BeWell^™^, which was intended to facilitate recovery, needs to be further evaluated.

## Introduction

The aim of primary health care in Sweden is to provide the population with basic healthcare, and is the first line of care, even for mental illnesses regardless of diagnosis and age [1]. Common mental disorders, e.g. anxiety, depression and stress-related exhaustion disorders, increase in societies globally [2]. These are now the most common diagnoses for sick leave in Sweden [3]. The present study is part of a project to evaluate a newly developed health-promotion intervention for people with stress-related exhaustion disorders. The photo-supported intervention, BeWell^™^, is intended for use as a treatment approach in primary healthcare services [4].

Stress-related illness can affect a person’s ability to work and increase the risk for sick leave [5], but can also affect social relationships [6]. Symptoms of stress can for the individual entail exhaustion and difficulties in concentrating and sleeping. These reduce the ability to manage activities in daily life, and deteriorate the experience of health and well-being when experienced as severe [7,8]. Self-care advice includes maintaining daily routines, socialising, taking part in recreational activities, and getting sufficient sleep with the aim of promoting health and well-being [9]. There are a number of non-pharmacological interventions for symptom relief and/or for promoting return to work that can be offered at Swedish primary care services when people with stress-related exhaustion disorders seek healthcare for support. These include cognitive behavioural therapy and multimodal rehabilitation [10], occupational therapy interventions [11,12], nature-based interventions [13,14], interventions focusing on work rehabilitation [15,16], and person-centred eHealth interventions [17]. The government in Sweden has recently proposed that patients are not only to be offered interventions aimed at reducing and alleviating symptoms, but also preventive and health-promotion interventions [1,18]. However, there is as yet limited evidence for the use of these types of interventions in primary care.

With the intention to ‘flip the coin’ towards health promotion, and not focus on symptom reduction, we have developed a new intervention, BeWell^™^ [4], derived from the findings of a previous project [19–21]. Women diagnosed with an exhaustion disorder were interviewed after having taken photographs of what well-being meant for them despite symptoms of stress. The findings showed that well-being implied a possibility of just being, to experience balance in everyday life and to be surrounded by a supportive environment. The participants said that they gained a new, more positive, perspective in their everyday life, and they asked for more training in this perspective [19,20]. BeWell^™^ aims to support the person to strengthen what is health-promoting and contributes to well-being in everyday life. The intervention is based on knowledge about health, well-being [22,23], occupations in everyday life [22,24], and visual methodology [25]. Furthermore, BeWell^™^ entails photo-supported conversations, based on the patients’ photographs on what he/she perceives to be related to their well-being. These photographs are used in the sessions as a support for the patient for reflecting about what contributes to their own well-being, despite suffering from an exhaustion disorder.

The BeWell^™^ intervention needs to be investigated before a possible implementation in primary healthcare services. The aim of this study was thus to evaluate self-reported health and well-being prior to and after a controlled clinical intervention with photo-supported conversations about well-being. The research question was:

Do patients’ health and well-being improve after the BeWell^™^ intervention in terms of exhaustion, depression and anxiety, sense of coherence (SOC), quality of life, occupational balance, and work ability?

## Materials and methods

This controlled clinical study has a quasi-experimental design [26], i.e. with no cluster randomisation or individual randomisation. Pre-test and post-test quantitative data were obtained from an intervention group and a control group completing questionnaires about health and well-being. The data were collected from January 2020 to December 2022. The study is part of a project about BeWell^™^ with mixed methods, described in detail in a study protocol [4], and registered as Clinical Trial (NCT04832295).

### Participants

Participants who met the inclusion criteria, age 20–67 years and diagnosed by their general practitioner with ‘Other reactions to severe stress’ ICD-10 F43.8 [27,28] or ‘Reactions to severe stress, unspecified’ F43.9 [27] were recruited. Exclusion criteria were concomitant severe somatic disease (symptoms of stress due to e.g. a cancer diagnosis), neurodevelopmental diagnosis (e.g. autism and/or attention deficit hyperactivity disorder), psychotic disorders, linguistic difficulties in Swedish and/or cognitive impairments that hindered comprehension when completing self-rating questionnaires.

### Context and procedure

The study took place in primary healthcare centres (PHCCs) in two regions in southern Sweden. A total of 16 PHCCs were contacted and 15 consented to participate. Pragmatic reasons, such as organisational and structural circumstances determined the PHCC management’s decisions about whether to participate in the intervention or control group. Six PHCCs participated in the intervention while nine centres chose to participate as controls. These two groups were comparable in terms of the size of the catchment area, urban and rural areas, socio-economic aspects of the PHCC. Furthermore, they were also comparable regarding resources and which interventions they could offer as care as usual, which were in line with what Swedish primary care usually offers. Presumptive participants who met the inclusion criteria, based on the diagnosis reported in the patient’s medical record, were asked by their healthcare professionals at the PHCC. If the patient gave a positive response the healthcare professional contacted the project manager. A researcher/research assistant then met each participant individually, and informed consent was obtained prior to data collection. The intervention was performed within ordinary primary care. All participating PHCCs received a symbolic compensation per recruited participant.

### Interventions

The participants in the intervention group received BeWell^™^ in addition to care as usual. The control group solely received care as usual. The healthcare professionals, who recruited the participants, met the research group regularly twice a year throughout the process, to discuss recruitment and the implementation of the study process. They also had the opportunity to discuss recruitment of participants with the first author (ABG) if necessary.

#### Care as usual

Care as usual, in this primary care context was described by the healthcare professionals who recruited the participants. The professionals completed a form, including possible interventions they usually offered the patients, i.e. care as usual. This generally included medical examination and assessment, drug prescription, physiotherapy, occupational therapy and supportive consultations or cognitive behavioural therapy with a supposed wide range of variation based on individual needs.

#### BeWell^TM^

The participating occupational therapists who carried out the BeWell^™^ intervention, were trained theoretically and practically prior to recruiting participants and carried out the intervention, as described in the study protocol [4].

**Figure 1.**
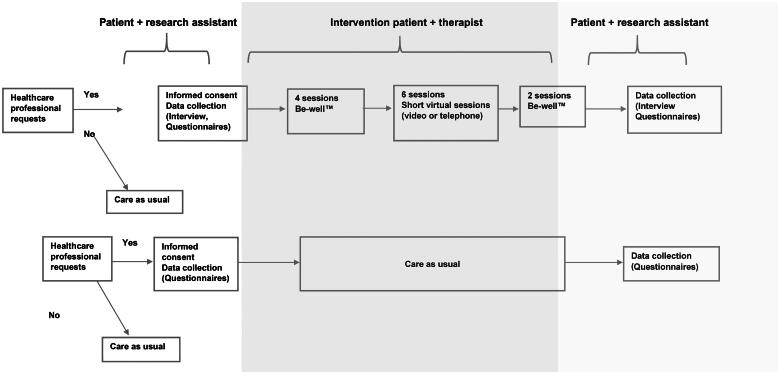
Treatment process.

Each participant in BeWell^TM^ met their occupational therapist a total of 12 sessions (Figure 1). The first four sessions were face-to-face meetings lasting 45–60 min and were carried out at the PHCC. Prior to each of these sessions, the patient took photographs on their mobile phones about what they considered to be related to their well-being, despite having an exhaustion disorder. The photographs were sent to the occupational therapist, who enlarged them to A4 format and brought them to the session. The photographs were used as a starting point for supportive conversations, in which the patient was encouraged to talk about, and find strategies to enhance their well-being in everyday life. The patient and occupational therapist then met for six short virtual sessions lasting 15–20 min with the aim of supporting and confirming the patient in their process towards increased well-being. The intervention was completed with two face-to-face sessions lasting 45–60 min and held at the PHCC; the first of these dealt with a ‘look in the rear-view mirror’ and based on all the photographs, the patient and occupational therapist reflected upon the former’s well-being. All the photographs were used in the final session in order to look forward and reflect on how to maintain the process about continuing to increase well-being in everyday life. The intervention was performed during the Covid-19 pandemic, although most of the face-to-face sessions were held as planned, while some of them were conducted virtually.

### Data collection

Two of the authors (ABG and UH), and one research assistant performed the data collection in the intervention group, and three other research assistants performed the data collection in the control group. The present study includes the quantitative data collected at baseline and after the treatment period, approximately three to six months after baseline. Each participant was asked about background data in a structured interview, i.e. age, gender, living status, education level, main occupation (working, studying, on sick leave), employment (public/private sector), level of sick leave/not sick leave, and physical activities, and they completed self-rating questionnaires focusing on health and well-being.

### Questionnaires

#### Primary outcome

Symptoms of exhaustion were measured with the Karolinska Exhaustion Disorder Scale (KEDS), in which exhaustion, cognitive problems, sleeping, and tolerance to stress are in focus [29]. KEDS consists of nine items, each scoring from 0 to 6 on an ordinal scale, the higher the score the greater the level of exhaustion. The separate scores are aggregated, with a cut-off of 19 (out of 54) for being considered in a risk zone for exhaustion. KEDS is commonly used in primary healthcare, and the Swedish version has shown diverse psychometric properties; from good [29] to limited [30].

#### Secondary outcomes

Symptoms of anxiety and depression were measured with the Hospital Anxiety and Depression Scale (HADS) [31]. The responses to the two sub-scales, depression (HADS-D) and anxiety (HADS-A) in HADS were assessed in the present study. HADS contains seven items for depression and anxiety respectively, scoring from 0 to 3 on an ordinal scale, the higher the score the greater severity of the symptoms. Scores from 11 or above indicated probable severe anxiety or depression. HADS is commonly used in primary healthcare, and the Swedish version has been found to be valid [32,33].

Quality of life, which deals with satisfaction with life (work, leisure, relationships, sexual health, and mental and physical health) was measured with the Manchester Short Assessment of quality of life (MANSA) [34]. MANSA has twelve items, scoring from 1 to 7 on an ordinal scale, the higher the scoring the greater the rating of quality of life, and the Swedish version has shown good psychometric properties [35].

SOC, which deals with stress resilience and includes the three components; comprehensibility, manageability and meaningfulness, was measured with the short 13-item SOC scale [36]. The scoring is from 1 to 7 on an ordinal scale, the higher the scoring the greater the level of SOC. The SOC-13, which can be related to experiences of health and well-being, is commonly used, and the Swedish version has shown good psychometric properties [37].

Occupational balance, which deals with satisfaction with the amount and variation of everyday activities, was measured with the Occupational Balance Questionnaire (OBQ11) [38]. OBQ11 has eleven items, scoring from 0 to 3 on an ordinal scale, the higher the scoring the greater level of occupational balance, and the Swedish version has shown sufficient psychometric properties [38,39].

Work ability, which deals with views of current and future employment and worker role, was measured with the Worker Role Self-assessment (WRS-18) [40]. WRS-18 has 16 items for those without any employment, and 18 items for those with an employment, scoring from 1 to 5 on an ordinal scale, the higher scoring the greater the assessment of the worker role. The Swedish version of the WRS-18 has shown good psychometric properties [41].

### Data analysis

A power calculation based on the primary outcome, change in exhaustion symptoms measured with KEDS, was performed. The standard deviation was assumed to be 8.74 based on a previous study [42] and a clinically relevant difference in the KEDS mean between intervention and control was anticipated to be seven points. At least 35 patients would be needed in each group to achieve 80% power with a statistical significance of *p* = .05.

Data quality was carefully checked and complemented when needed, and there was thus no missing internal data. Five participants in the intervention group and five participants in the control group discontinued their participation in the study and did not contribute any post-treatment data. No imputations were made. The analyses were conducted using IBM SPSS Statistics 27.0. Non-parametric methods were used for crude analysis as the data was based on nominal and ordinal scales. Chi-squared tests were used to compare background characteristics between the intervention and control groups and the Mann Whitney U-test (Wilcoxon rank sum test) was used to analyse unpaired independent groups of numerical data while the Wilcoxon signed rank test was applied for paired data. Linear regression models were used to investigate the impact of the intervention on the primary outcome variable adjusted for KEDS and sick leave at baseline, and group affiliation (intervention or control). KEDS data were considered sufficiently normally distributed for analysis.

## Results

**Figure 2.**
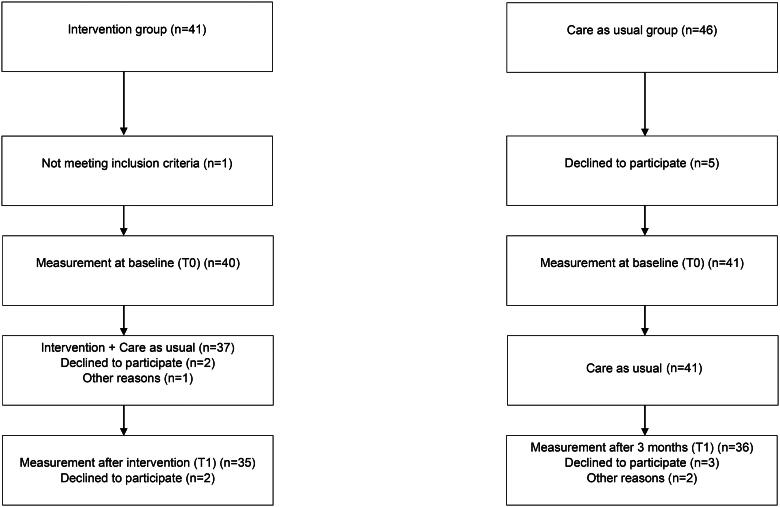
Flowchart of the participants’ recruitment and participation.

Eighty-one patients participated at baseline, and 71 completed post-treatment data (Figure 2). The median age of the participants at baseline was 46 years, with a range from 21 to 63 years. There was no difference in age between the intervention group (median 47 years, inter quartile range (IQR) 35–55) and the control group (median 44 years, IQR 36–55) (*p* = .784). There were no significant differences at baseline between the intervention group and the control group concerning gender, living status and education (Table 1). A larger proportion of the participants in the intervention group were, at baseline, on sick leave (67% vs 24%, *p* < .001) and the participants in the intervention group who were on sick leave were more often on full time sick leave. Significantly fewer participants in the intervention group performed physical training weekly (37% vs 61%, *p* = .046).

**Table 1.** Background characteristics of the primary care study population with exhaustion disorders.

	Intervention (*n* = 40)	Control (*n* = 41)	
	n (%)	n (%)	*p* ^a^
Gender			.547
Male	7 (17.5)	5 (12.2)	
Female	33 (82.5)	36 (87.8)	
Living status			.112
Married/with partner	32 (80.0)	26 (63.4)	
Alone	6 (15.0)	14 (34.1)	
Other	2 (5.0)	1 (2.4)	
Highest completed education level			.298
Primary and lower-secondary school	2 (5.0)	0	
High school	24 (60.0)	22 (53.7)	
University	14 (35.0)	19 (46.3)	
Main occupation			<.001
Work	12 (30.0)	28 (68.3)	
Study	1 (2.5)	2 (4.9)	
On sick leave	27 (67.5)	10 (24.4)	
Other	0	1 (2.4)	
Employment			.294
Public sector	25 (62.5)	21 (61.8)	
Private sector	8 (24.2)	13 (31.7)	
Sick leave			<.001
Full time	28 (70.0)	11 (26.8)	
Part time	8 (20.0)	17 (41.5)	
No sick leave	4 (10.0)	13 (31.7)	
Physical training weekly			
Yes	15 (37.5)	25 (61.0)	.046
No	25 (62.5)	16 (39.0)	

^a^ Based on two-sided exact Chi^2^-test.

There were no significant differences between the intervention group and the control group at baseline in terms of any of the self-rated questionnaires (KEDS *p* = .146, HADS-A *p* = .951, HADS-D *p* = .440, SOC-13 *p* = .723, MANSA *p* = .261, OBQ11 *p* = .928 and WRS-18 *p* = .609). Significant improvements between baseline and post-treatment were found for both groups for all outcome measures of health and well-being except for work ability where the improvement was more modest. The improvements in health and well-being were similar in the two groups, however, the participants in the intervention group improved their occupational balance and work ability, while it was unchanged in the control group (Table 2).

**Table 2.** Measures of health and well-being at baseline and post-treatment in the two groups.

	Intervention				Control				Difference between groups
	Baseline (*n* = 40) Md (IQR)^c^	Follow-up (*n* = 35) Md (IQR)	Difference Md (IQR)	*p* ^d^	Baseline (*n* = 41) Md (IQR)	Follow-up (*n* = 36) Md (IQR)	Difference Md (IQR)	*p* ^d^	*p* ^e^
Primary outcome^a^									
KEDS	34.0 (29.2–37.7)	22.0 (16.0–27.0)	−9.0 (−14.0 to −0.4)	<.001	30 (23.5–36.0)	23.5 (18.2–32.7)	−4.0 (−11.0 to −1.0)	<.001	.035
Secondary outcomes^b^									
HADS-A	10.0 (8.2–14.0)	8.0 (6.0–10.0)	−3.0 (−4.0 to −1.0)	<.001	11.0 (8.0–14.0)	8.0 (5.0–10.7)	−3.0 (–5.0 to −0.2)	<.001	.963
HADS-D	9.0 (6.2–12.0)	5.0 (3.0–8.0)	−2.0 (−7.0–0)	<.001	9.0 (5.0–11.0)	6.5 (3.2–9.0)	−2.0 (–4.0–0)	.002	.391
MANSA	51.5 (44.5–56.0)	55.0 (46.0–65.0)	5.0 (–1.0–10.0)	.002	53.0 (47.5–59.0)	57.5 (48.5–63.5)	3.0 (–2.7–8.0)	.020	.300
SOC	52.2 (44.2–62.7)	59.0 (52.0–68.0)	5.0 (1.0–8.0)	<.001	49.0 (45.0–62.0)	59.5 (51.0–65.7)	5.0 (1.2–12.5)	<.001	.747
OBQ11	10.5 (7.0–15.0)	15.0 (11.0–20.0)	3.0 (0–7.0)	.004	11.0 (7.0–15.5)	11.0 (8.0–15.7)	1.0 (–1.0–3.0)	.122	.141
WRS-18	50.0 (45.0–57.5)	54.0 (47.0–61.0)	3.0 (–2.2–8.2)	.045	51.5 (42.2–57.0)	54.0 (44.5–57.0)	1.0 (–5.0–4.0)	.707	.232

^a^ Karolinska Exhaustion Disorder Scale (KEDS).

^b^ Hospital Anxiety and Depression Scale, anxiety subscale (HADS-A), and depression subscale (HADS-D), Manchester Short Assessment of quality of life (MANSA), sense of coherence (SOC), Occupational Balance Questionnaire (OBQ11), Worker Role Self-assessment (WRS-18).

^c^ Median and interquartile range.

^d^ Based on two-sided related samples Wilcoxon signed rank test.

^e^ Based on independent samples Mann–Whitney U-test.

The average difference in KEDS points between baseline and after the treatment period was minus six points. The reduction of symptoms of exhaustion (primary outcome) was larger in the intervention group than in the control group (median difference on KEDS −9.0 vs −4.0, *p* = .035) (Table 2). Adjusted linear regression revealed that the size of the difference in KEDS was associated with KEDS at baseline, the higher the baseline KEDS score, the larger the reduction (one more point at baseline KEDS corresponded to almost 0.5 point larger reduction). The median difference in KEDS was not associated with group affiliation (intervention or control) or with proportion of sick leave at baseline. There were no differences in the median score in a comparison between the intervention group and the control group at follow-up for any of the secondary outcomes assessing health and well-being.

## Discussion

### Main findings

Stress-related symptoms decreased considerably among the participating primary care patients over the treatment period in this quasi-experimental clinical intervention study. There were similar improvements in health and well-being irrespective of treatment according to care as usual or with the addition of the novel BeWell^™^ method with photo-supported conversations.

### Discussion of results

The participants in this study, a majority of whom were women currently on sick leave, were diagnosed with a stress-related illness. They thus reflect a representative sample of primary care patients in working ages in accordance with public data from the Swedish National Board of Health and Welfare and the Swedish Social Insurance Agency [43,44]. Most of the participants were on sick leave, had an employment, and were frequently employed in professions that entailed contact with people in the public sector. These occupations have been associated with a higher risk of suffering from stress-related illness in Sweden [44]. The participants in this study scored high levels of KEDS at baseline, while their scores for symptoms of anxiety and depression were rather low, which are outcomes in line with what can be expected in a study population with stress-related illness [45].

There were improvements for all outcomes in the intervention group. The participants’ self-rated health and well-being improved; in terms of decreased symptoms of exhaustion, anxiety and depression, and an improved quality of life, SOC, occupational balance and work ability. There were similar improvements in the control (care as usual) group, apart from the variables occupational balance and work ability, where a significant improvement was not reached. However, there were no differences between groups over time. Symptom improvement over time is in line with the findings in a recent study including sick-listed patients with stress-related illness in primary care [46]. The difference found in the present study between the groups might be related to the baseline difference in rates of sick leave. It is conceivable that ratings of occupational balance and work ability did not change to the same extent as more participants in the control group worked. However, the findings that occupational balance and work ability improved significantly only in the intervention group might also be an indication that the intervention addresses these components to a greater extent than symptom reduction. This is in line with the health promotive idea of the BeWell^TM^ concept [4] in supporting patients to reflect on their own well-being and coping, despite suffering from stress-related illness. This interpretation is also in agreement with findings from a qualitative study exploring the therapists’ experiences of the outcomes of the BeWell^TM^ [47]. BeWell^™^, is based on theories on well-being [23] and occupations in everyday life [22], as well as occupational balance [24], and visual methodology [25]. The finding that the intervention group increased their occupational balance more, from a median score of 10.5 to a median score of 15, supports the theoretical concept of BeWell^™^ [4], and could be seen as promising. The scores can be compared with those in a cross-sectional study by Lexén et al. [48], in which occupational therapists in employment scored their own occupational balance with a median of 13. Furthermore, another possible explanation for the improvements in self-rated occupational balance only occurring in the intervention groups might be due to this being a common focus within occupational therapy in general [49].

There were, however, no differences in outcomes between the groups, with the exception of symptoms of exhaustion, measured with KEDS. These results showing no differences between the groups, resemble findings reported in previous studies investigating similar interventions with controls [12,16,50]. Participants with stress-related illness received the rehabilitation ‘Redesigning Daily Occupations’ (ReDO) or care as usual in a study by Eklund and Erlandsson [12], where both groups improved significantly, but no significant differences were found between the groups. Similarly, our results are in line with studies including participants with common mental disorders; participants received the Tree Theme Method^®^ or regular occupational therapy with best practice in a study by Gunnarsson et al. [50], and participants in a pilot study by Danielsson et al. [16] received work-directed rehabilitation or physical activity. The experiences of the participants in qualitative interviews in the latter study of the benefit of the treatment were such that their belief in their capacity increased, and the importance of a person-centred approach was highlighted [51]. The results of these studies taken together show that the participants improved their ratings and seemed to have benefitted from their treatment, irrespective of whether they received the intervention or care as usual. This indicates that a variety of interventions are needed with a person-centred approach [52,53]. The novel intervention BeWell^™^ can be one such intervention, adding to the range of options of interventions with a health-promoting focus.

### Methodological considerations

The strengths of this study are that it was a well-controlled clinical trial with a high quality of data, few drop-outs and no internal missing data due to the rigorous control of the research implementation. There was close contact between the clinical staff performing recruitment and treatment of patients and the researchers. A study protocol was published prior to the study implementation and all questionnaires applied to measure outcomes had previously been validated, psychometrically tested and established in research studies [4]. Another strength is the application of a control group receiving care as usual as recommended for research in clinical context [54,55]. Care as usual was defined as the treatments for patients with stress-related illness available at the recruited PHCCs, and included various treatment methods provided for patients with common mental disorders and stress-related illness in ordinary primary care in Sweden.

A power calculation was performed in an early phase of the project to determine the size of the study groups for detecting a change in the primary outcome, a reduction in the KEDS score between groups. A sufficient number of participants were recruited to correspond to this calculation. However, the baseline differences hindered the possibilities for making comparisons as the two study groups varied greatly in terms of the proportion of patients on full-time sick leave. A possible explanation for this might be that the professionals who recruited patients for the control PHCCs generally asked patients about participating at an earlier stage of stress-related illness than occurred for the intervention group thus leading to larger proportion still being in work. Sick leave is partly an indication of the severity of an illness, but other factors related to daily life at home and at work are important with regard to return to work [56]. Even if there was no randomisation procedure, the participants were recruited based on the same inclusion and exclusion criteria. The fact that the intervention group were more often on sick leave at baseline might indicate longer period of illness or more severe illness. People with stress-related ill health often seek primary care long before being put on sick leave is in question [57,58]. There is no baseline data available on how long time the participants in the current study had been on sick leave prior to inclusion, which is a limitation.

The study was pragmatic and carried out in a real-world clinical context [59] of primary care, in contrast to many studies performed in other specialised clinical settings, which contributes to a high level of external validity. The external validity may be limited by the Swedish primary care setting and also the predominance of women. The gender distribution, however, mirrors the clinical situation with more women seeking healthcare for stress-related disorders and common mental disorders [60]. This study shows that it was feasible to perform the BeWell^TM^ intervention as an additional treatment in primary care and to assess post-treatment measurements for a relatively large study group after treatment from baseline. This indicates that it is possible to implement evaluations of non-pharmacological interventions [61]. The inclusion of a clinical control group, although not randomised, contributes to the research field with empirical data regarding the patient group and care as usual in primary care. Moreover, this study illustrates the practical difficulties related to management and resources involved when conducting clinical research in ordinary primary care. The overall aim of the research project [4], of which this study was a part, is the feasibility of the BeWell^TM^ intervention in primary care and the experiences of participating patients and staff [47], which will be presented in forthcoming reports. If the overall feasibility of the intervention is assessed to be on a high level then this might support the implementation of a randomised controlled trial of its treatment effect, which includes a suitable study population.

## Conclusion

This study found that patients with stress-related exhaustion disorders, both those receiving BeWell^™^, and those receiving care as usual improved their ratings of health and well-being considerably after a treatment period of three to six months. Comparison between the groups is uncertain as they differed at baseline regarding rates of sick leave. Follow-up studies with a longer perspective, as well as findings from qualitative interviews will enhance the understandings of the feasibility of the novel BeWell^TM^ treatment as a health promoting and person-centred approach for stress-related disorders.

## Ethical approval

This study was approved by the Regional Ethical Board in Linköping, Sweden (Dnr 2019-04334; 2020-03148), and was performed in accordance with the principles stated in the Declaration of Helsinki [62]. The participants were given information about the study aim, and what it would entail to participate in this study prior to giving their informed consent. They also received information about confidentiality, voluntariness, the opportunity to withdrawn at any time without any explanation, and the utilisation requirement. All participants provided written informed consent to participate in this study.
